# An improved extraction method for surface dosage of insecticides on treated textile fabrics

**DOI:** 10.1186/s12936-016-1647-1

**Published:** 2017-01-04

**Authors:** Florence Dieval, Jérémy Bouyer, Jean-François Fafet

**Affiliations:** 1 Laboratoire de Physique Et Mécanique Textile, ENSISA Werner, 11 rue Alfred Werner, 68093 Mulhouse, France; 2 CIRAD, UMR15 CIRAD-INRA Control of exotic and emerging animal diseases, Campus international de Baillarguet, 34398 Montpellier, France

**Keywords:** Textile, Effective surface concentration, Insecticide, Mechanical extraction, Dosage, Permethrin, Chromatography, Bioavailable

## Abstract

**Background:**

Tens of millions of people live in mosquito-infested regions and controlling mosquito-borne diseases is one of the major interventions aimed at alleviating poverty worldwide. The use of insecticide-treated textiles is one of the most widespread control measures. This includes bed nets, battle clothing or, more generally, textiles use for clothing. These textiles are generally treated with permethrin as active ingredient, which is dosed after extraction of the active molecule present throughout the fabric (measured in mg permethrin/g of fabric) and does not take the effective concentration on the textile surfaces into account. The objective of this study was to propose an improved dosage method that enables measurement of the bioavailable or effective part of active ingredients on the surface of textile treated with insecticides.

**Methods:**

The proposed method relies on mechanical extraction of active molecules on the surface of the textile in direct contact with either the skin or with the targeted arthropod.

**Results:**

The results showed that the amount of permethrin measured using the current method is about 200 times higher than the effective surface concentration of the insecticide. In addition, the type of weave or knit influences the effective concentrations of permethrin on the surface of the textile. With the current dosage method, the variation in the concentration of permethrin depending on the type of weave is maximum 8%, whereas with the proposed method, it varies by about 50%. These results were confirmed by bioassays, in which the type of weave significantly affected (p < 10^−3^) the 100% knockdown time of *Anopheles gambiae*.

**Conclusions:**

The bioefficacy of insecticide treatments of fabrics is directly correlated with the effective concentration of insecticide on the textile surface, which can be quantified using the method proposed. This improved method could be used to redefine the limits of actual concentrations of active substance after assessment of the bioefficacy of the treatment and the risk to human health. Further, it enables assessments of the kinetics of insecticide migration in the case of long-lasting insecticide treatment.

## Background

As part of vector control and particularly in the fight against malaria, in 1955, the World Health Organization (WHO) recommended indoor residual spraying (IRS) of insecticides as part of the Global Malaria Eradication Programme [1]. IRS consists of spraying wall surfaces, and the WHO defined different surface concentrations to be used depending on the insecticides used and the different materials to which it was applied. Today, these concentrations are still expressed in mg of active substance per m^2^ to be sprayed.

In the late 1970s, the WHO recommended the use of insecticide-treated nets (ITNs). Like for IRS, the WHO defined the mg of active substance per m^2^ of mosquito net treated as the concentration unit [2]. Many authors [3, 4] who sought to establish a relationship between bioefficacy of treated fabrics and the surface concentration of the insecticide, used a protocol defined by the WHO as a guideline for measuring the activity and chemical composition of nets [5]. Some methods were also published by the Collaborative International Pesticides Analytical Council (CIPAC) for LLINs (long-lasting insecticidal nets) [6]. These methods enable estimation of the total insecticide content (mass concentration) and the results are expressed in grams of active ingredient per gram, or in percent as well as in milligrams of active ingredient per square metre of textile. The following formula is used to obtain the insecticide surface concentration:

$${\text{Surface concentration }}({\text{mg}}\, {\text{m}}^{ 2} = {\text{ Mass concentration }}\left( {{\text{mg}} \,{\text{g}}^{ - 1} } \right) \times {\text{Mass of textile material per unit area }}\left( {{\text{g}} \,{\text{m}}^{ - 2} } \right)$$

**Fig. 1:**
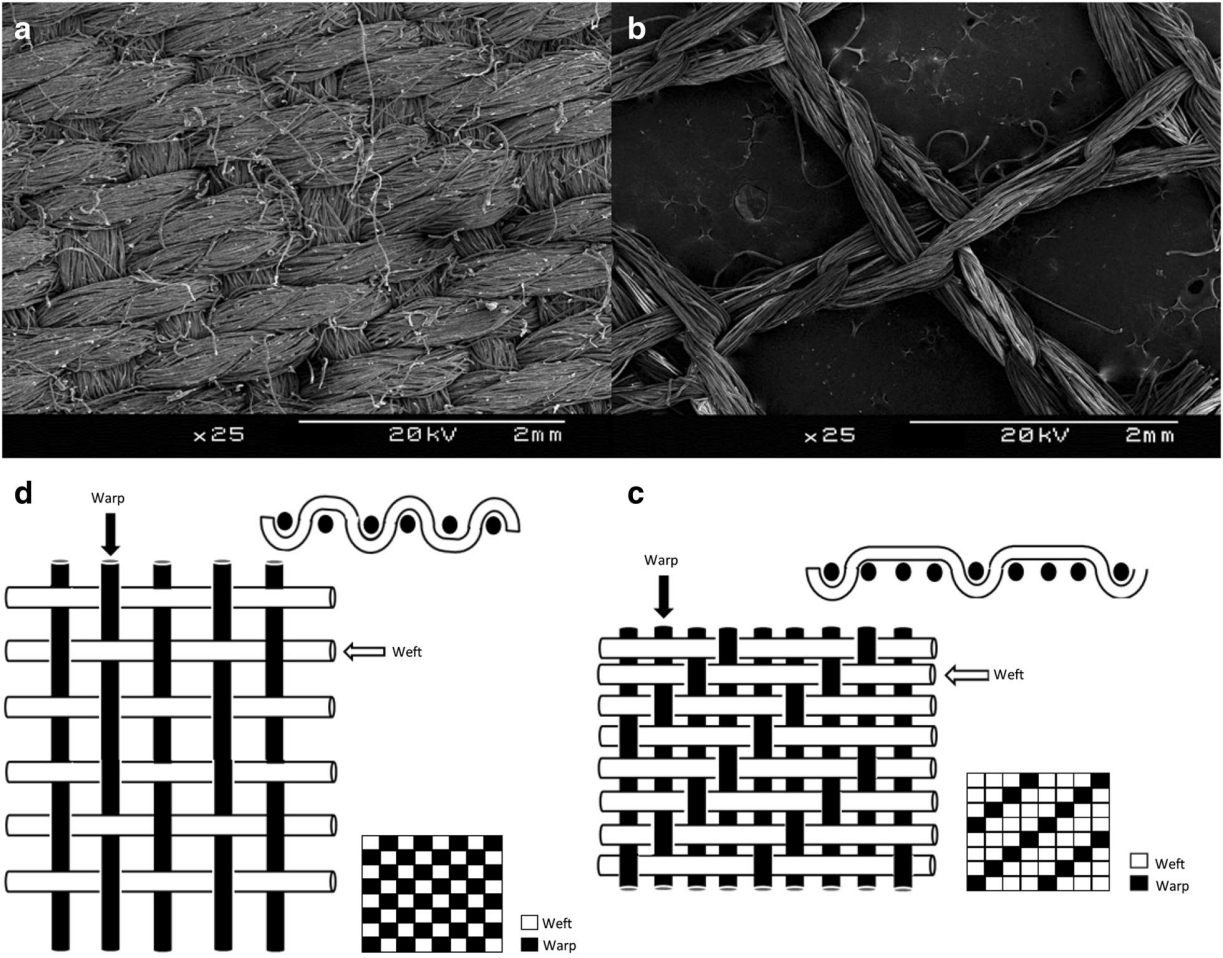
Structures of various fabrics impacting the surface availability of insecticides. **a** *Upper left* a woven textile scanned with an electronic microscope, **b** *upper right* a knit textile scanned using an electronic microscope, **c** *bottom right* schema of the interlacing structure and weave of a twill fabric and **d** *bottom left* schema of the interlacing structure and weave of a plain weave fabric

IRS treatment differs from textile treatments because each support has a different structure (Fig. 1). The textile structure of a woven fabric differs from that of a knitted textile. A textile is created by intersecting a set of yarns. The way the threads interlace with each other is called the weave. In woven fabrics, the yarns are always straight and parallel lengthwise (warp threads) or in the direction of the width (weft threads), whereas the thread of a knitted fabric follows a meandering path, forming symmetrical loops or meshes. The main characteristics of all fabrics are thickness, the structure of the yarn, the density and kind of interlacement.

All textiles are different. If the insecticide treatment of a mosquito net (weighing around 50 g/m^2^) is compared with that of a cloth (weighing around 210 g/m^2^), the effective amount of insecticide on the surface of each textile will be different. If the textile is a mosquito net, the same insecticide dosage per square metre means that the amount of insecticide on the surface yarn is higher than on a continuous textile such as a woven cloth. Mosquitoes touch the textile at discrete points with the tip of their six legs and, if the net has a surface representing (for example) 5% of a tightly woven textile, the concentration to which the mosquito is exposed is 20 times higher on the yarn of a net than on the yarn in a woven textile.

In the CIPAC protocol defining the dosage of permethrin content in LLIN [6], a method is described to assess the surface concentration of permethrin and is calculated in µg of permethrin per g of mosquito net (µg/g). This concentration is the effective amount of permethrin on all yarns comprising the mosquito net sample and on all sides of the yarns.

Green et al. [7] used a mechanical method of extraction with a special device that makes rectilinear rubbing cycles (back and forth). In this method, the insecticide is mechanically extracted from one side of a 12 cm^2^ area, so the rubbing cannot extract the insecticide in all directions of the treated textile surface.

In the following, a improved method is proposed to measure the effective surface concentration of insecticide on insecticide-treated textile including mosquito netting and fabrics that also uses a mechanical extraction method (by rubbing) of insecticides but which differs from that of Green et al. in the larger area of rubbed surface and multidirectional extraction.

This improved method makes it possible to measure the effective surface concentration on only one side of the treated textile. The method was tested on a LLIN (Olyset Net™) and four fabrics treated by impregnation using a padding system for textile finishing.

## Methods

### Fabric samples

**Fig. 2:**
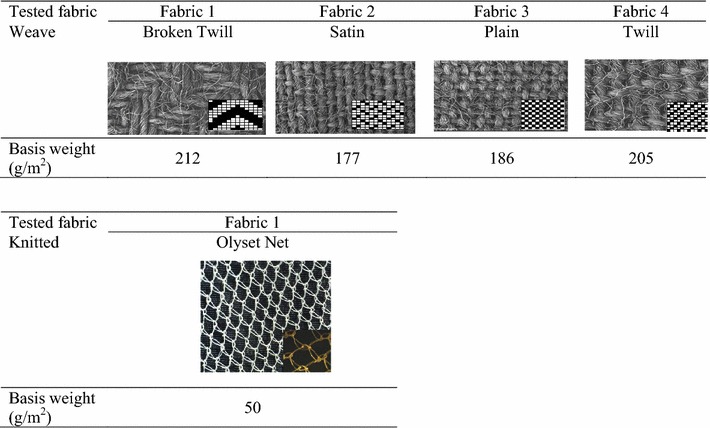
Structure and weight of the tested fabrics

To study the effect of the structure of the fabric, four woven 100% carded cotton (plain, twill, satin, and broken twill) samples and one mosquito net (knitted fabric) 100% High density Polyethylene (Olyset net™ already treated with 2% permethrin) were selected (Fig. 2).

### Treatment of samples with permethrin

The samples of textile were treated by padding at constant speed using a laboratory padder. Three samples of each fabric structure (30 cm × 100 cm corresponding to 0.3 m^2^) were treated.

Only the cis isomer of permethrin is effective. In the case of Olyset™, the permethrin used has an isomer cis/trans ratio of 40:60. This means that out of the 2% mass of permethrin contained in Olyset™ fibers, only 8 mg of cis isomer is contained per g of knitted fabric (40% of 20 mg permethrin/g knitted fabric). As 40/60 permethrin is not available in Europe, 25/75 permethrin was used to treat the other fabrics. To use the same amount of cis isomer as that used in Olyset™, 3.2% of 25/75 permethrin equivalent was used on the fabrics (1.6 times more than Olyset™). In addition, considering the adsorption capacity of fabrics after impregnation, which is about 75% (0.75 l of bath adsorbed per kilogram of fabric), a ~4% more concentrated bath of permethrin was used.

Four hundred grams of dipping bath was prepared for each sample treated. The bath contained 80 g/l of 50% EC permethrin formulation (emulsifiable concentrate EC 500 N from Envirochem Europe SAS—FR), with an isomeric ratio of 25:75 (25% cis isomer and 75% trans isomer), 3 g/l ULTRAVON CN (surfactant—Huntsman Textile Effects—CH). Each sample was immersed for 2 s and the excess solution was removed between two rollers (pressure of two bars), then the samples were dried in a domestic tumble dryer for 45 min at a maximum temperature of 75 °C.

### Determination of total content of active ingredient

High performance liquid chromatography (HPLC) analysis was performed between 48 and 72 h after treatment of the samples. An uncut sample of 50–100 mg (named “Mt” and expressed in mg of treated fabric) was placed in a glass vial equipped with a tight stopper containing 5 ml (named "v1” and expressed in ml) of analytical grade methanol (ROTISOLV^®^, LC–MS). Permethrin was removed from the fabric by sonication at 35 kHz at room temperature in a SONOREX DIGITEC ultrasonic compact bath for 45 min.

The solution was then analysed an Agilent 1100 Series HPLC equipped with a UV diode array detector (DAD) and a column NUCLEODUR ^®^ 100-5-C18 −5 μm, Ø 4.6 mm, inside length 250 mm.

Beforehand, a permethrin calibration curve defined the area under the absorbance peak as a function of the permethrin concentration (mg/l). The permethrin concentration (Cp) in mg/l of extraction solution (v1) was determined using this calibration curve. The quantity of permethrin (Qp), expressed in mg, extracted from the treated textile fabric was calculated as follows:

$${\text{Qp}} = {\text{v1}} \times {\text{Cp}}/ 1000 \, \left( {\text{quantity in mg}} \right)$$

The weight/weight concentration of active ingredient of the treated samples was then calculated as follows:

$${\text{Permethrin mass concentration }}\left( {{\text{in}}\,\% \,{\text{W}}/{\text{W}}} \right) = 100 \times {\text{Qp }}\left( {\text{mg of permethrin}} \right)/{\text{Mt }}\left( {\text{mg of treated fabric}} \right)$$

### Determination of surface concentration of permethrin

Martindale machines are normally used to determine the abrasion and pilling resistance of all kinds of textiles including woven, non-woven, and knitted fabrics. Samples are pulled taut and loaded onto the lower plates of the Martindale machine. Small discs of worsted wool or wire mesh (the abrading material) are continually rubbed at low pressures against the textile samples in a Lissajous figure (oscillating cycle).

**Fig. 3:**
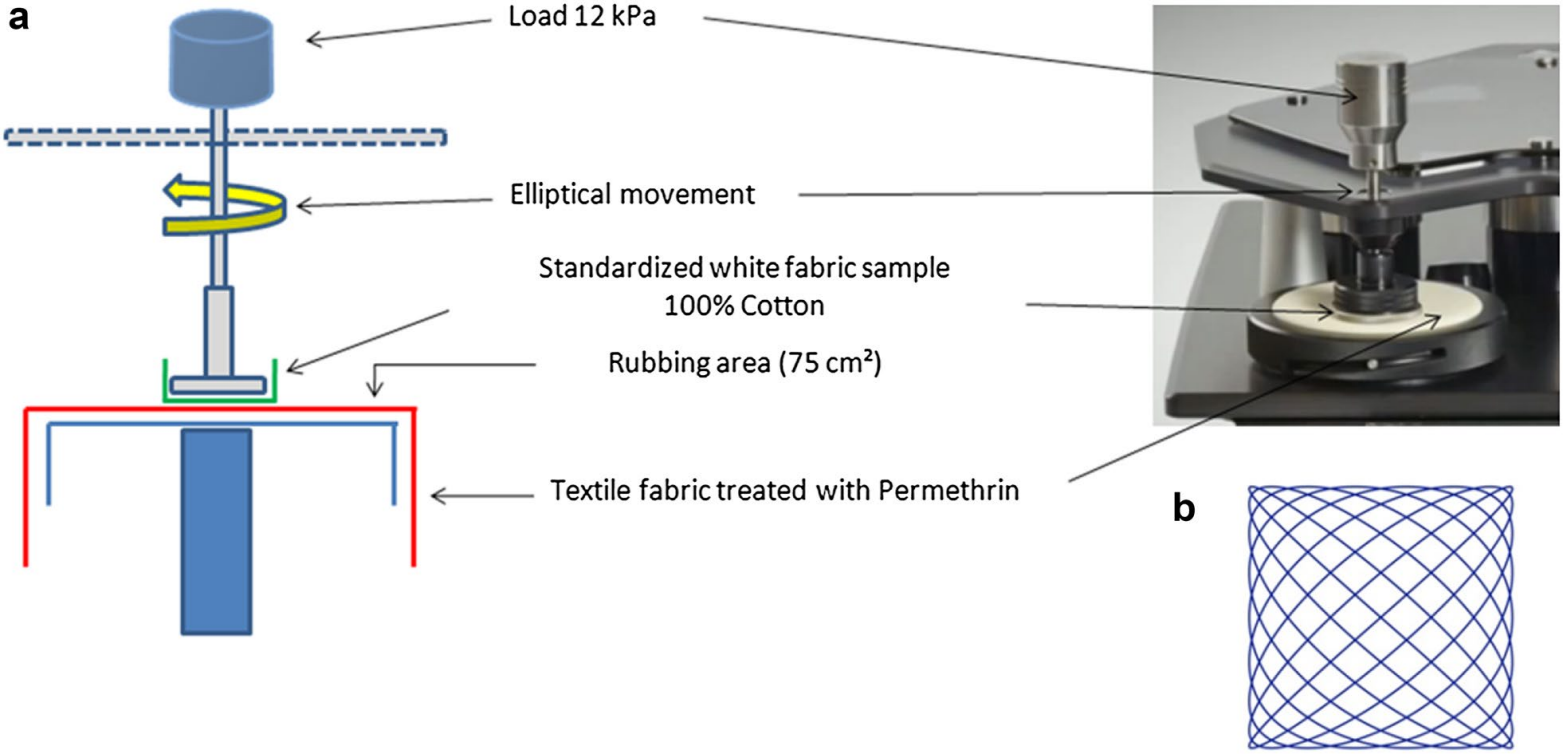
Martindale machine (**a**) and the Lissajous figure performed by this machine (**b**)

The Lissajous figure traced by the Martindale machine is drawn in continuously changing directions (Fig. 3). It is complete after each 16th Martindale cycle. Here the Martindale machine (James Heal Martindale Model 905 with 5 abrasion stations) was used to capture the available permethrin at the surface of the treated fabrics. This mechanical extraction of permethrin was thus not made by abrasion but by rubbing. A sample of treated fabric with a 140 mm diameter was cut with a special device and fixed on the rubbing area (Fig. 3).

Using the pressing weight provided with the Martindale, the treated fabric sample was laid flat and held in place on the rubbing area by clamps. With a second circular cutting apparatus, a piece of non-abrading standard cotton fabric (used in tests of colour fastness to rubbing ISO 105-X12) with a 38 mm diameter was cut and used as rubbing pad. This standardized cotton sample was placed in the centre of the sample holder and was topped with a piece of polyurethane foam with a diameter of 38 mm. The whole was clamped onto the sample holder. A weight of 12 kPa was then added on the sample holder. The entire sample holder (with the load) was placed on the rubbing area of the sample of treated fabric. The number of rubbing cycles was set using the control panel of Martindale machine. During the rubbing cycles, surface permethrin is transferred from the different textile samples to the cotton sample mounted on to the rubbing pad.

Permethrin was extracted 48 h after treatment and drying. The standardized cotton samples were recovered after 32 rubbing cycles (two total Lissajous patterns), 128 rubbing cycles (8 total Lissajous patterns), 512 rubbing cycles (32 total Lissajous patterns) and 2048 rubbing cycles (128 total Lissajous patterns).

### Determination of permethrin collected by rubbing on standardized cotton fabric samples

Each recovered standardized cotton fabric sample was placed individually in a glass vial equipped with a sealing plug. Permethrin was removed from the fabric using methanol as solvent (50 ml Methanol ROTISOLV^®^, LC–MS-Grade for each sample) and by sonication for 45 min at room temperature. The resulting solution was analysed by HPLC and the quantity of permethrin recovered (in mg) from each standardized cotton fabric sample was determined. The quantity of permethrin was extracted from a 75 cm^2^ rubbed surface. The effective surface concentration of permethrin was calculated as follows: quantity of permethrin recovered (mg)/75 cm^2^.

**Fig. 4:**
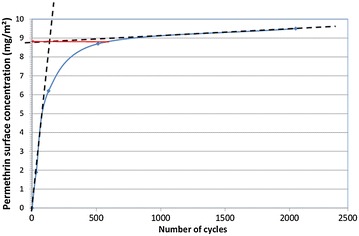
Determination of effective permethrin surface concentration in mg/m^2^

The results are expressed in mg of permethrin per square metre of treated fabric. A curve “permethrin surface concentration in mg/m^2^ (Y axis) versus the number of rubbing cycles (Y axis)” was drawn for each treated fabric and the effective surface concentration of permethrin (in mg/m^2^) was determined using a tangent method (Fig. 4). The reproducibility of the method was tested. For each test, an average of three assays was performed (Table 1). Three curves were plotted from three sets of specimens. The maximum surface concentration was determined for each curve. Average values, standard deviations and variation coefficients (CV) were then calculated.

**Table 1:** Reproducibility of the test results on broken twill fabric

Sample	Number of cycles			
	32	128	512	2048
	Permethrin concentration in mg/m^2^ after 32 rubbing cycles	Permethrin concentration in mg/m^2^ after 128 rubbing cycles	Permethrin concentration in mg/m^2^ after 512 rubbing cycles	Permethrin concentration in mg/m^2^ after 2048 rubbing cycles
1	1.9	6.2	8.7	9.5
2	1.9	6.3	8.4	8.7
3	1.9	6.4	7.9	8.7
Average value	1.9	6.3	8.3	9.0
Standard deviation	0.0	0.1	0.3	0.5
Variation coefficient (%)	0.0	1.3	4.0	4.2

### Bioassays

Insecticidal activity was tested using a protocol similar to the method named median knock-down time (MKDT) [8]. The mosquito species used was *Anopheles gambiae* (Kisumu strain) continuously reared colonies. The mosquitos were obtained from the ATRC Laboratory (Arusha, Tanzania). A temperature of 27 °C and 70% RH was maintained throughout the study. Adults were fed with 10% sucrose solution, and only non-blood-fed, 2–5 day old female mosquitoes were used for testing.

Four series of 15 mosquitoes were placed under a glass cover in a circular chamber (10 cm in diameter and 1 cm in height) cut in a Plexiglas plate. Mosquitoes were continually exposed to the treated fabrics. The period of exposure necessary to obtain a 100% knockdown of (knockdown time 100%—KDT 100) was measured. Following the definitions of the WHO, Knockdown was defined as inability to move/migrate (maximum exposure time 60 min). Twenty-four hours after exposure, mortality and functional mortality were determined (female mosquitoes with 3 or less legs were considered as “functionally dead”).

KDT100 was analysed using mixed linear models in which the type of fabric was used as a fixed effect and the repeat was used as a random effect [9]. Two other models were built in which the surface concentration of permethrin and the total concentration of insecticide were used as fixed effects instead of the fabric type. The corrected Akaïke information criteria (AICc) was used to compare models [10].

## Results

### Permethrin concentrations

All “permethrin surface concentration in mg/m^2^ as a function of the number of rubbing cycle” curves showed asymptotic behavior with first, an ascending part corresponding to the extraction of the permethrin directly available on the surface of the treated fabrics and an asymptotic part corresponding to the extraction of the permethrin present inside the textile structure. The inflection point corresponded to the first steps of the fabric abrasion and the mechanical extraction of the first textile fibers containing permethrin.

Tables 1 and 2 give the results of the reproducibility of the test dosage on the broken twill fabric and Olyset™ mosquito net. In view of the quantities extracted, the reproducibility of the method is very good.

**Table 2:** Reproducibility of the test results on Olyset™

Sample	Number of cycles			
	32	128	512	2048
	Permethrin concentration in mg/m^2^ after 32 rubbing cycles	Permethrin concentration in mg/m^2^ after 128 rubbing cycles	Permethrin concentration in mg/m^2^ after 512 rubbing cycles	Permethrin concentration in mg/m^2^ after 2048 rubbing cycles
1	4.0	8.0	12.0	14.2
2	3.1	8.2	12.1	14.8
3	4.2	7.8	11.8	14.0
Average value	3.8	8.0	12.0	14.3
Standard deviation	0.5	0.2	0.1	0.3
Variation coefficient (%)	17.7	2.0	1.0	2.4

The results in Table 3 show that the quantity of permethrin determined by the total extraction method (mass concentration, which is the current method) is on average, 200 times greater than the surface concentration determined using the improved method (effective surface concentration of insecticide) for woven fabrics and 83 times for Olyset™. There was an influence not only of the type of weave on the real concentration of permethrin, but also a difference between woven and knitted fabrics. Indeed, with the current dosage method, the difference in the concentration of permethrin depending on the type of weave was maximum 8% (6459 mg/m^2^ on plain fabric versus 5957 mg/m^2^ on satin fabric), whereas it varied from 50% in the proposed method (25 mg/m^2^ on twill and satin fabric versus 50 mg/m^2^ on broken twill fabric).

**Table 3:** Differences in permethrin concentrations depending on the method

Weave	Permethrin mass concentration (%)	Fabric weight g/m^2^	Permethrin surface concentration mg/m^2^ with current method (1)	Permethrin surface concentration mg/m^2^ with improved method (2)	Ratio between both method (1/2)
Broken twill	2.92	212	6193	50	124
Satin	3.37	177	5957	25	238
Plain	3.46	186	6459	32	201
Twill	2.97	205	6073	25	242
Olyset	2.01	50	1000	12	83

### Bioassay results

**Fig. 5:**
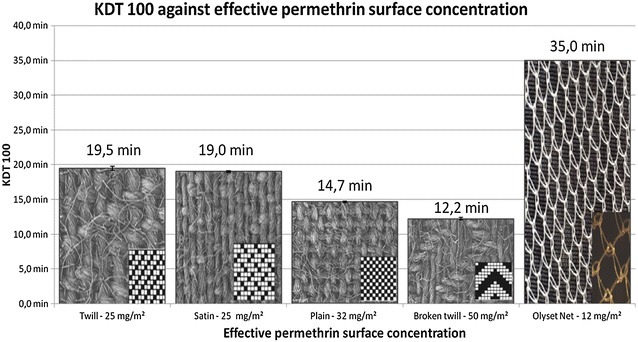
Relationship between KDT100 and the surface concentration of permethrin

Table 4 presents the results of the bioassays. Whereas the knock down and mortality rates were similar in all the samples, there was quite marked variability in the time needed to reach a knockdown rate of 100% (KDT_100_), also shown in Fig. 5. This time can be considered as an inverse measure of the biological efficiency (the longer, the less efficient). The maximum KDT_100_ was obtained for Olyset™ (32.2 min, SD 0.6), followed by the twill fabric (19.5 min, SD O.3), the satin fabric (19.0 min, SD 0.1), then the plain (14.7 min, SD 0.1) and broken (12.2 min, SD 0.2) fabrics which were all much more efficient than Olyset™ (p < 10^−3^). All comparisons were highly significant, even between the twill and satin fabrics (p = 0.04) (Fig. 5; Table 4). Both surface concentrations improved the predictions comparing the use of KDT _100_ and the use of the permethrin mass concentration only as a fixed factor (AICc of 121.7). However, using the effective permethrin surface concentration calculated using the improved method in the model enhanced the latter much more than the effective permethrin surface concentration calculated using the standard method (AICc of 79.5 instead of 114.2), demonstrating the usefulness of this method for predicting biological efficiency. However, it should be noted that these models still did not fit the data as well as the model using the type of fabrics, suggesting that other factors than the surface concentration of the insecticide are involved in biological efficiency, for example, the type of contact between the tarsus and the fabrics.

**Table 4:** Results of the bioassays

	Total number of mosquitoes tested	KDT_100_ (min.) (a)	Number of mosquitoes KD	Number of mosquitoes dead 24H	Number of mosquitoes functionally dead	% KD	% Mortality 24H (b)	% Functional mortality (c)
Control								
1	16	60	0	1	0	0	6.25	6.25
2	15	60	0	0	0	0	0	0
3	15	60	0	0	0	0	0	0
4	15	60	0	1	0	0	6.67	6.67
Total	61	60	0	2	0	0	3.28	3.28
Broken twill—49 mg/m^2^								
1	14	12.08	14	13	1	100	92.62	100
2	15	12.11	15	12	3	100	79.32	100
3	13	12.35	13	12	1	100	92.05	100
4	15	12.42	15	14	1	100	93.11	100
Total	57	12.24	57	51	6	100	89.12	100
Satin—25 mg/m^2^								
1	14	19.02	14	13	1	100	92.62	100
2	14	19.16	14	14	0	100	100	100
3	16	18.95	16	16	0	100	100	100
4	11	19	11	11	0	100	100	100
Total	55	19.03	55	54	1	100	98.12	100
Plain—32 mg/m^2^								
1	14	14.56	14	13	0	100	92.62	92.62
2	15	14.63	15	14	1	100	93.11	100
3	15	14.74	15	15	0	100	100	100
4	15	14.81	15	15	0	100	100	100
Total	59	14.69	59	57	1	100	96.5	98.25
Twill—25 mg/m^2^								
1	16	19.58	16	16	0	100	100	100
2	17	19.8	17	17	0	100	100	100
3	14	19.4	14	13	1	100	92.62	100
4	15	19.16	15	15	0	100	100	100
Total	62	19.49	62	61	1	100	98.15	100
Olyset—12 mg/m^2^								
1	13	34.4	13	13	0	100	100	100
2	18	35.62	18	17	1	100	94.4	100
3	16	35.15	16	15	1	100	93.8	100
4	11	35.53	11	11	0	100	100	100
Total	58	35.17	58	56	2	100	97.05	100

(a) KDT100 (min.): The time frame needed to obtain 100% knockdown of mosquitoes constantly exposed to permethrin treated fabrics. Mortality was recorded in two ways

(b) % 24 h Mortality: counting the mosquitoes really dead after 24 h (real mortality)

(c) % Functional mortality: including surviving mosquitoes with three or less legs after 24 h

## Discussion

The marked differences in surface concentration between the current method and the proposed method can be explained by the fact that in the current method, all the extracted active substance is related to only one surface which is considered as perfectly smooth and free of pores. However, a fabric is a 3D structure whose thickness, porosity, and specific surfaces differ depending on the type of textile treated, as demonstrated by the results obtained in this study. The improved method revealed this effect much more clearly, even though there was still a significant impact of the type of fabric that was not related to the surface concentration.

In the context of vector control, it is thus not the total quantity of insecticide present in the textile structure that is important but the bioavailable fraction on the surface. Moreover, the effective fraction of insecticide on the surface of the fabric not only makes it possible to characterize the bioefficacy of the treatment against arthropods (on the outer surface of clothes), but also to know how much insecticide comes into contact with the skin and sweat (on the inner surface side of clothes) and that has toxicological effect, i.e. the amount of insecticide really in contact with the skin of the person wearing the treated item).

**Fig. 6:**
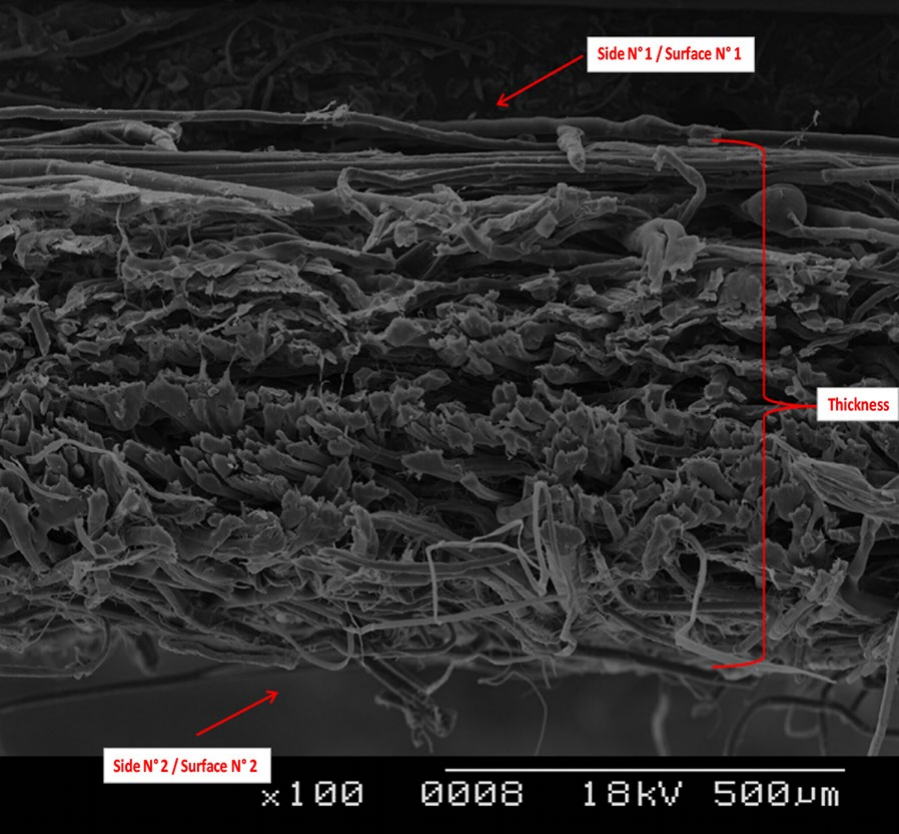
Thickness of a woven textile fabric observed by scanning electron microscopy

The cross section of a woven textile fabric analysed by scanning electron microscopy helps understand the textile structure and especially the importance of the thickness of the fabric (Fig. 6). When a textile is treated with an insecticide, the active molecules spread throughout the textile structure, not only remain on the surface. This part of the insecticide has two major implications in terms of bioefficacy and risks of toxicity associated with the use of treated fabrics.

The accurate calculation of the amount of active ingredient present only on the surface of the fabric is an important aspect that should be taken into account in risk assessment. To be even more accurate and to systematically obtain a satisfactory level of bioefficacy (which depends on the bioavailability of the active ingredient), this part of the insecticide should not be determined based on mass concentration (mg/m^2^ or g/kg) but on the effective surface concentration of the insecticide.

According to the WHO criteria, the maximum permethrin concentration authorized for textile treatment is 1300 mg/m^2^, which is the concentration also used by militaries. Based on the results of the tests, and mass concentration (mg/m^2^ or g/kg), all the treated fabrics tested in this study should be considered as potentially toxic and their sale prohibited. However according to the improved method of measuring the surface concentration of insecticide, in reality, none of these fabrics are cytotoxic to humans.

Concerning bioefficacy, according to the criteria used by armies, the minimum permethrin efficiency is obtained with a concentration of 200 mg/m^2^.

However, as shown in tests on four fabrics treated with about 3% permethrin (30 g/kg of fabric), these fabrics contain a dose that is five times higher than the maximum permitted dose and 31 times higher than the minimum recommended by the WHO or by armies according to the current method (mass concentration), and are therefore considered effective. But according to the improved method described here, textiles with a surface concentration of between 25 and 50 mg/m^2^ should be inefficient (because lower than the current recommendations).

For Olyset™ net, the improved method determined an effective surface concentration of only 12 mg/m^2^ compared to 1000 mg/m^2^ using the classical method and around 30 mg/m^2^ using the CIPAC method (surface concentration and release index). The actual measured concentration was thus 17 times lower than the minimum concentration recommended by the WHO or armies.

These results help understand the variations in bioefficacy on textile fabrics treated with insecticide. They provide important information not only for the control of malaria vectors, but also of other vector species like tsetse flies, for which the use of insecticide-impregnated targets and traps [11], or impregnated nets around pig or cattle nets [12, 13] are widely used control methods [14].

This improved method and all the protocols used in this study are in accordance with the PCT Patent N° WO/2012/175820 entitled “Method of determining the surface concentration of active molecules of a surface of an active element and device for the implementation thereof” [15].

The American Association of Textile Chemists and Colorists (AATCC), in its AATCC Committee RA49, reported that this improved method will soon become a new official standard titled “Determination by Extraction of Free Insecticide Available on Textile Surface”.

## Conclusion

Current insecticide dosage methods do not enable measurement of the concentration of effective active ingredient present (i.e. bioavailable) on the surface of the impregnated fabrics. All current methods are based on total insecticide extraction measured by weight (mass concentration) and do not allow accurate prediction of bioassays compared to the effective surface concentration of insecticide. Regarding toxicological risks and especially dermal exposure, assessments of risk related to the use of treated fabric are overestimated.

The present article presented a improved method to measure the effective insecticide available on all types of fabrics treated with insecticides (non-woven, mosquito nets, clothes, battle dress, curtains, plastic sheeting, etc.). The method is recommended for all materials treated with insecticides, by incorporation or by coating, and allows better prediction of the bioefficacy of the fabrics.

The analytical dosage described here may also help evaluate the migration kinetics of insecticides (after washing and drying) to the surface of the treated materials and help measure the effective insecticide concentration once equilibrium is reached. The first results were obtained on Olyset™ at a temperature of 22 °C, in the initial state (unwashed) and after washing at 30 °C (according to the washing procedure describe in the WHO Guidelines for laboratory and field testing of long-lasting insecticidal mosquito nets) and will be the subject of a new publication.

These results underline the need to reconsider the present assays of active substances on treated fabrics and to redefine the limits of concentrations of efficient insecticides depending on the type of fabric. The relationship between the bioefficacy and the concentration of active ingredients and different types of fabrics with different characteristics require further investigation.
